# Health is not just the absence of disease …

**DOI:** 10.1093/ije/dyw063

**Published:** 2016-05-11

**Authors:** Alarcos Cieza, Cornelia Oberhauser, Jerome Bickenbach, Richard N Jones, Tevfik Bedirhan Üstün, Nenad Kostanjsek, John N Morris, Somnath Chatterji

**Affiliations:** ^1^ Faculty of Social and Human Sciences, University of Southampton, Southampton, UK,; ^2^ Department of Medical Informatics, Biometry and Epidemiology, LMU Munich, Munich, Germany,; ^3^ Swiss Paraplegic Research, Nottwil, Switzerland,; ^4^ Department of Health Sciences and Health Policy, University of Lucerne, Lucerne, Switzerland,; ^5^ Departments of Psychiatry and Human Behavior and Neurology, Warren Alpert Medical School, Brown University, Providence, RI, USA,; ^6^ Classification, Terminology and Standards, Department of Health Statistics and Informatics, World Health Organization, Geneva, Switzerland,; ^7^ Hebrew Senior Life’s Institute for Aging Research, Social and Health Policy Research, Boston, MA, USA and; ^8^ Multi-Country Studies, Department of Measurement and Health Information Systems, World Health Organization, Geneva, Switzerland

We thank Stuckler and Reeves for their commentary ^1^ on our re-evaluation ^3^ of claims made by Banks *et al.*^4^ and others that the English are healthier than the US Americans. Living in England, Stuckler and Reeves may be forgiven for concluding their commentary by saying ‘So should you live in the US or England? Judging on the health data alone, we find the weight of evidence still (slightly) favours—England’. The point of our article was not to suggest that people move countries, but rather to propose a better methodology for tackling the difficult public health challenge of comparing health across populations.

They ^1^ begin by claiming that our aim was to operationalize the well-known World Health Organization (WHO) 1948 definition of health ^2^ . Our aim was actually the very different one of arguing that it is a mistake to adopt the Banks *et al.*^4^ understanding of health merely as the absence of disease. We rather claim that health needs to be measured as a vector of functioning in a parsimonious set of domains that matches the intuitive notion of health such that one can compare the health of people with (for example) diabetes and those with depression, an approach proposed by Salomon *et al.*^5^

We read with pleasure when Stuckler and Reeves ^1^ point out the convergence of inferences obtained by examining recent Global Burden Disease (GBD) 2010 efforts ^6^ and our own. We agree on this. The difference between us is that whereas the GBD says that the health differences between the USA and the UK are trivial, we say they are really, really trivial! To see this, consider their Table 1, ^1^ in which healthy life expectancy (HALE) at age 50 is reported to be about 25 years for both the UK and the USA, with a 0.4-year advantage for the UK. If healthy survival after age 50 has a Poisson distribution, this difference amounts to 8% of a standard deviation. Life expectancy (LE), by contrast, at age 50 is about 31–32 years for the UK and the USA, with a 0.8-year advantage for the UK. If survival after age 50 has a Poisson distribution, this difference amounts to 14% of a standard deviation. But these differences are indeed very, very small. Imagine a sample of 1000 persons aged 50 years and older from the UK and a matching number from the USA. Taking all possible pairs, selecting one person from the UK and one from the USA, if we predicted a longer life expectancy for the person from the UK we would be right 52% of the time, instead of the 50% of the time that might be expected if the life expectancy distributions were identical in the USA and UK or by chance. Our Rasch-based health metric, ^3^ on the other hand, suggests a smaller difference (about 2% of a standard deviation) and implies a correct guess about health in 51% of pairs of USA/UK elders instead of the 50% expected if the distributions were identical.

A related feature of our approach, ^3^ which Stuckler and Reeves ^1^ call ‘a major limitation’, is our reliance on self-report data, and the fact that culture is an important driver of how respondents answer questions about their health. In fact, our results argue for diminishing differences between the USA and the UK after these cross-national differences in self-reporting of health are adjusted. In other words, our results are based on the Rasch model, in which the health score is estimated after correcting for differential item functioning (DIF) and therefore accounting for reporting biases and hence population invariant. We think that culture and, more broadly, all social and environmental differences between the USA and the UK, influence self-reported health. The Rasch and DIF scoring procedure—with different thresholds for different sex, age and national groups—is an attempt to address this.

Although we do not report it, had we done our Rasch scoring of health without correction for DIF by national group, we might have had larger USA/UK difference in health. This means that it is possible that all of the (neglible) health differences between the USA and the UK may be due to measurement error caused by cultural differences in the self-reporting of health.

Regarding the ‘curious reporting conventions’, at least one is the convention of this journal, namely that of reporting 90% rather than 95% confidence intervals for the main result, following Sterne and Davey Smith. ^7^ As for the suggestion that the goodness-of-fit tests we used would not permit our conclusion, this would be quite correct if we had based our conclusion on these tests. In fact, we only used these tests as further supporting evidence for our main conclusions, which were derived from the regression coefficients resulting from the linear additive model as reported in Table 4 of our paper. ^3^ Another concern of theirs ^1^ is that although we object to Banks *et al.*^4^ looking only at a few health conditions, we, it would seem, reduce all health conditions to a single unidimensional scale. But this is to misconstrue the fundamental difference between ‘counting diseases’ as a measure of severity (like counting apples and oranges and then deciding which of these two groups are sweeter overall) and constructing a metric of health based on the functioning domains that are constitutive of the essence of health. Finally, we are told that we have constructed a ‘straw man’ ^1^ by citing examples of where the Banks *et al.*^4^ conclusions about UK health advantage have been relied on. It suffices to invite readers to peruse the Institute of Medicine Report *U.S. Health in International Perspective: Shorter Lives, Poorer Health* (2013) ^8^ which cites Banks *et al.* , and similar studies, extensively.

Finally, although we are reluctant to recommend that US Americans immigrate to the UK to improve their health, we definitely would recommend, when comparing the health of populations, to supplement comparing prevalence of diseases with a more nuanced and rich analysis based on a fuller conception of health, since after all, health is more than the absence of disease…

**Conflict of interest:** None.
